# Total and caspase-cleaved cytokeratin 18 in chronic cholecystitis: A prospective study

**DOI:** 10.1186/1471-230X-8-14

**Published:** 2008-05-06

**Authors:** Constantinos Simopoulos, Alexandra K Tsaroucha, Byron Asimakopoulos, Alexandra Giatromanolaki, Paschalis Gavriilidis, Alexandros Polychronidis, Anastasios Karayiannakis

**Affiliations:** 1 2nd Department of Surgery, Medical School, Democritus University of Thrace, 68100 Alexandroupolis, Greece; 2 Laboratory of Physiology, Medical School, Democritus University of Thrace, 68100 Alexandroupolis, Greece; 3 Department of Pathology, Medical School, Democritus University of Thrace, 68100 Alexandroupolis, Greece

## Abstract

**Background:**

Cell death mode has been studied in cancer, autoimmune, and neurodegenerative diseases. In this study, apoptosis and necrosis are investigated for the first time in patients with chronic calculous cholecystitis.

**Methods and materials:**

Thirty five (35) patients (27 women and 8 men, aged 55.65 ± 13.48 years) with symptomatic chronic calculous cholecystitis underwent laparoscopic cholecystectomy. The early specific apoptotic tendency (caspase-cleaved cytokeratin 18) was studied in these patients with M30 Apoptosense ELISA and the total cytokerarin 18 (both derived from apoptosis and necrosis) with M65 ELISA. The ratio M30/M65 (caspase-cleaved to total cytokeratin 18) was also computed. According to the histopathological examination, the patients were divided in two groups: group A included patients with chronic inactive cholecystitis (n = 10), and group B those with chronic active cholecystitis (n = 25).

**Results:**

The concentrations of caspase-cleaved cytokerarin 18 (CK18), and especially those of total CK18, were higher in bile samples than in serum samples. In group B, there were significant differences between serum and bile samples regarding both caspase-cleaved CK18 and total CK18. Cells staining positive for caspase-cleaved CK18 were present in the epithelial cells of the mucosa of the gallbladder.

**Conclusion:**

CK18 is expressed in the gallbladder epithelial cells. The concentrations of both caspase-cleaved CK18 and total CK18 were higher in bile samples than in serum samples. The levels of total CK18, as well as caspase-cleaved CK18, do not seem to differ between active and inactive chronic cholecystitis.

## Background

Cytokeratins (epithelial keratins) are an important component of the intermediate filament system. They are mainly insoluble molecules playing an important role in cellular mechanics (cell shape, motility, division and cell-cell contact). There are two types of cytokeratins: a) Type I (9–20) keratins that are relatively acidic and bearing a small molecular weight (40–56.5 kDa), and b) Type II (1–8) that are relatively basic-neutral of larger molecular weight (53–67 kDa) [1]. Proliferating cells have a substantial pool of two soluble cytokeratins, namely CK8 and CK18, and their concentration is high during the G2-M phase of the cell cycle [2]. During apoptosis, CK18 are cleaved by caspases at position Asp396 producing relatively stable fragments [3-8]. These fragments can be detected in cells, sera and other tissue fluids being a biomarker of apoptosis, while soluble intact CK18 may be released during both necrosis and apoptosis. In this way, the ratio of fragment/intact CK18 (i.e., caspase-cleaved to total CK18) may be a helpful tool in quantifying apoptosis and necrosis during various pathological conditions.

In an attempt to study the process of cell death in the gallbladder epithelium of patients with chronic cholecystitis and cholelithiasis, we examined the concentration of CK18 and its neo-epitope M30 released after CK18 caspase-mediated cleavage in the sera and bile of these patients. Furthermore, immunohistochemistry was performed to confirm the presence of apoptotic CK18 fragments in the gallbladder epithelium.

## Methods

### Patients

This prospective study was performed at the University Hospital of Alexandroupolis, 2^nd ^Department of Surgery, Medical School, Democritus University of Thrace. Thirty five (35) patients (27 women and 8 men, aged 55.65 ± 13.48 years) suffering from chronic calculous cholecystitis were included. Four gallbladder epithelium control samples were also added in the study. The samples were from patients not suffering from chololithiasis and cholecystitis. All patients underwent laparoscopic cholecystectomy. The patients signed a written consent and did not receive any monetary compensation for participating in the study. The study was approved by the Democritus University Ethics Committee.

Serum and bile samples were collected before any manipulation of patients. The serum samples were collected pre-operatively. The bile samples were collected after trocar entry. All samples were aliquoted and kept frozen at -75°C for further analysis. Tissue of the gallbladder of all patients was received and was sent to the pathologist. Detection of apoptosis was performed in formalin fixed and paraffin embedded tissue sections from the gallbladder samples with immunohistochemistry. The gallbladder specimens were classified according to the histopathological report in two groups: group A included patients with chronic inactive cholecystitis (n = 10), and group B those with chronic active cholecystitis (n = 25).

### Measurements of CK18 in serum and bile samples

In serum and bile samples, the caspase-cleaved CK18 and the total CK18 were detected by commercially available ELISA kits. Caspase-cleaved CK18 was measured with M30-Apoptosence assay (Peviva, Bromma, Sweden). This assay uses a mouse monoclonal antibody detecting a neo-epitope, only formed upon caspase-cleavage of CK18 at position Asp396. Therefore, values obtained by M30-Apoptosence assay represent the apoptotic cell death. Total CK18 was measured with M65 ELISA kit (Peviva, Bromma, Sweden), which uses two mouse monoclonal antibodies specific for conventional epitopes on CK18. Thus, values obtained with M65 represent the total cell death (both apoptosis and necrosis). The ratio of the values obtained by M30 and M65 represent the ratio of apoptosis to total cell death.

Since both ELISA kits were not validated for bile samples, validation tests were run in order to determine if the kit reagents are suitable for this type of samples. Namely, for M65 ELISA kit, a "recovery" experiment was designed as follows: A well mixed bile sample was diluted 1:4 and split into two aliquots. One aliquot was run as "neat sample". The other aliquot was spiked with the high standard (2000 U/l): 40 μ of the high standard were mixed with 160 μ of the bile sample – this was the "spiked sample". A "spiked control" was also prepared by mixing 15 μ of the high standard with 60 μ of diluent. These samples were measured and the recovery rate was determined with the formula: [(spiked sample – neat sample)/spiked control] – 100. This experiment was repeated three times with three different bile samples. The recovery rates were 136–155%. Furthermore, serial dilutions of the spiked samples were prepared: 1:2, 1:4 and 1:8. The parallelism of the diluted spiked samples to the standard curve was satisfactory.

For M30 ELISA kit, the parallelism to the standard curve was checked with multiple dilutions of three bile samples: 1:2, 1:4, 1:8, 1:16. The samples were diluted with 0 standards. There were satisfactory results for the dilutions 1:4, 1:8 and 1:16.

For the ELISA measurements, an automatic washing system for microplates (Multiwash Plus, TRI Continent, NY, USA) and a microplate reader at 450 nm (Anthos, Labtec Instr., Salzburg, Austria) were used. All samples were run in duplicate. Bile samples were diluted 1:4 or 1:8 with the appropriate diluent.

### Detection of caspase-cleaved CK18 in the epithelium of the gallbladder

The presence of caspase-cleaved CK18 was investigated in gallbladder tissue sections with M30 CytoDeath Monoclonal Antibody (Peviva, Bromma, Sweden) for all 35 patients. Sections were deparaffinised, placed in antigen unmasking buffer pH 6.0 (Trilogy, DAKO, Denmark) and microwaving was followed (3 × 4 min). Peroxidase was quenched with methanol and H_2_O_2 _3% for 15 minutes. The primary antibody (concentration 2 μg/ml/1:50) was applied overnight at room temperature. The Envision kit (DAKO, Denmark) was used for the subsequent steps. The color was developed by 15 min incubation with DAB solution and sections were weakly counterstained with hematoxylin. Normal rabbit immunoglobulin-G was substituted for the primary antibody as the negative control, at the same concentration as the primary antibody. The immunoreactivity of the M30 CytoDeath antibody was assessed in the cytoplasm of the epithelial cells of the gallbladder mucosa.

### Statistical analysis

Statistical analysis included descriptive statistics and comparisons between studied groups. As studied parameters did not follow the normal distribution (tested with Kolmogorov-Smirnov test for normality), non-parametric statistical tests were used. Comparisons between groups were performed with Mann-Whitney U test. Comparisons between serum and bile samples within groups were performed with Wilcoxon matched pairs test. The two-tailed significant level was set at p < 0.05. The software used for statistical analysis was STATISTICA 6.0 (StatSoft Inc., Tulsa, OK, USA). Values are given as mean ± standard deviation.

## Results

CK18 in both forms, total and caspase-cleaved, was detected in all samples of both groups. The levels of the two forms of CK18 in groups A and B in both the serum and the bile samples are presented in Table 1. There were no significant differences between the two groups.

**Table 1:** Caspase-cleaved CK18 (U/l), total CK18 (U/l) and their ratio in serum and bile samples.

		**Group A**	**Group B**	**Two-tailed p**
Serum	Caspase-cleaved CK18 (U/l)	292.550 ± 192.121	207.356 ± 139.373	0.231
	Total CK18 (U/l)	579.100 ± 372.171	431.286 ± 183.045	0.257
	Ratio: Caspase-cleaved CK18/total CK18	0.581 ± 0.173	0.583 ± 0.503	0.130
Bile	Caspase-cleaved CK18 (U/l)	963.280 ± 308.69	1490.571 ± 1045.862	0.186
	Total CK18 (U/l)	3292.360 ± 3041.213	5305.875 ± 2177.843	0.160
	Ratio: Caspase-cleaved CK18/total CK18	1.319 ± 2.288	0.598 ± 1.083	0.770

Values are presented as mean ± standard deviation. The comparisons were performed by Mann-Whitney U-test.

In general, the concentrations of both caspase-cleaved CK18 and total CK18 were higher in bile samples than in serum samples. In group B, the differences between serum and bile samples were significant regarding both caspase-cleaved CK18 (Wilcoxon matched pairs test: Z = 3.296, p = 0.001) and total CK18 (Wilcoxon matched pairs test: Z = 2.981, p = 0.003).

**Figure 1:**
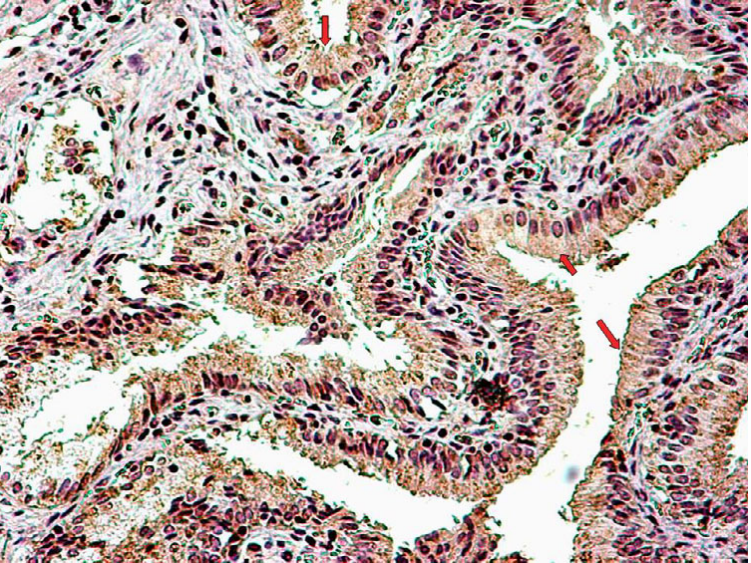
Detection of apoptosis in a formalin-fixed and paraffin-embedded tissue section of gallbladder sample showing confined cytoplasmic staining for caspase-cleaved CK18 with the use of M30 CytoDeath Monoclonal Antibody (Peviva, Bromma, Sweden).

**Figure 2:**
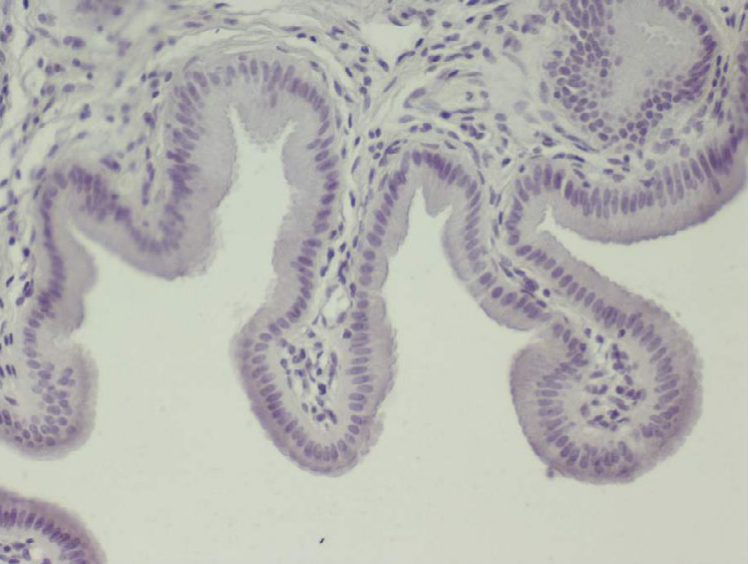
Tissue section of gallbladder sample negative for immunostain M30 × 200.

The relatively high levels of both caspase-cleaved CK18 and total CK18 detected in the bile compared to the serum, led us to use immunohistochemistry examination, with the monoclonal antibody M30 CytoDeath, to detect apoptosis (caspase-cleaved CK18) in the epithelial cells of the mucosa of the gallbladder. Figure 1 shows the staining of the cytoplasm of the epithelials cells of the mucosa of the gallbladder. Cells staining positive for caspase-cleaved CK18 were present in the epithelial cells in the group of chronic active cholecystitis. M30 was not expressed in acalculous gallbladder epithelia from four patients not suffering from cholecystitis (Figure 2).

## Discussion

The presence of increased caspase-cleaved CK18 serum levels in cancer patients has been recently presented, and seems to be a useful diagnostic marker for these patients [7,9-12]. Increased caspase-cleaved CK18 serum levels were found in primary and recurrent breast cancer patients, and in cancer patients with a large number of organs involved [10]. Increased serum levels of caspase-cleaved CK18 were also associated with trauma patients, patients with viral and autoimmune hepatitis of the liver, and liver transplant patients [13-17]. Therefore, the possibility that apoptosis may generate circulating products detectable in the serum and/or in other body fluids has an important diagnostic potential. In addition, there is an increasing interest on the diagnostic potential of both forms of CK18 (i.e., caspase-cleaved and total) in various, other than cancer, diseases, since the ratio of the two forms of CK18 seems to determine reliably the proportion of apoptosis within the total cell death [18-20]. Different studies showed that serum levels of M30-antigen and M65-antigen may be of clinical usefulness to identify patients with liver disease [21-23].

In this study, for the first time, the presence of both forms of CK18 in the bile of calculous cholecystitis patients was investigated. Caspase-cleaved CK18 was also studied in the gallbladder epithelium of these patients. Comparison was made between the levels of both caspase-cleaved CK18 and total CK18 in the bile and the serum, in order to see if apoptosis in the gallbladder of patients with calculous cholecystitis is a local phenomenon. Although, significant differences between the two groups of the study were not detected, it was shown that both forms of CK18 can be detected in bile of the patients with cholecystitis. Higher concentrations of both caspase-cleaved CK18 and total CK18 were measured in bile samples compared to serum samples, suggesting that cell death is prominent in the gallbladder, and that apoptosis and necrosis are local phenomena. The results of the immunohistochemical examination also confirmed apoptosis, with presence of caspase-cleaved CK18 in epithelial cells of the gallbladder.

In a previous study, Yanagisawa et al. examined the proliferative and apoptotic processes in patients with chronic cholecystitis [24]. Reactive proliferation of epithelial cells was noted using the Ki67 proliferation index, accompanying a clear apoptotic tendency of the gallbladder mucosa, confirmed by the intense p21WAF1 protein expression, a down-stream protein of the activated wild-type p53 gene, which is a key step of the apoptotic process [25]. This finding is in full accordance with our observation that CK18 apoptotic fragments are present in the gallbladder mucosa, bile and sera of patients with active cholecystitis. On the other hand, no apoptosis was observed in acalculous non-cholecystitis gallbladder epithelia.

## Conclusion

CK18 is expressed in the gallbladder epithelial cells and in the bile. The concentrations of both caspase-cleaved CK18 and total CK18 were higher in bile samples than in serum samples. The levels of total CK18, as well as caspase-cleaved CK18, do not seem to differ between active and inactive chronic cholecystitis.

## Competing interests

The authors declare that they have no competing interests.

## Authors' contributions

CS, AKT and AK designed the study. BA performed the ELISA measurements. AG performed the immunohistochemistry of tissue sections. CS, AKT, PG and AP were the surgical team and collected the samples. All authors have contributed to, read and approved the final version of the manuscript.

## Pre-publication history

The pre-publication history for this paper can be accessed here:


